# Does Using Indocyanine Green Fluorescence Imaging for Tumors Help in Determining the Safe Surgical Margin in Real-Time Navigation of Laparoscopic Hepatectomy? A Retrospective Study

**DOI:** 10.1245/s10434-022-12893-3

**Published:** 2022-12-09

**Authors:** Xinran Cai, Haijie Hong, Wei Pan, Jiangzhi Chen, Lei Jiang, Qiang Du, Ge Li, Shengzhe Lin, Yanling Chen

**Affiliations:** 1 Department of Hepatobiliary Surgery and Fujian Institute of Hepatobiliary Surgery, Fujian Medical University Union Hospital, Fuzhou, China; 2 Fujian Medical University Cancer Center, Fuzhou, China; 3 Key Laboratory of Ministry of Education for Gastrointestinal Cancer, Fujian Medical University, Fuzhou, China; 4 Fujian Key Laboratory of Tumor Microbiology, Department of Medical Microbiology, Fujian Medical University, Fuzhou, China

## Abstract

**Background:**

This study aims to investigate whether indocyanine green (ICG) tumor imaging helps determine the safe surgical margin in laparoscopic hepatectomy.

**Patients and Methods:**

Eighty-six patients with hepatic malignancies [including hepatocellular carcinoma (HCC) and colorectal liver metastasis (CRLM)] were included in this study. ICG-R15 testing was performed 5–7 days before surgery. Fluorescence staining of the tumor was detected by a fluorescent laparoscope, and the width of fluorescence band surrounding tumor was measured by an electronic vernier caliper.

**Results:**

The positive rate of hepatic malignant lesions successfully stained by ICG fluorescence was 96.0% (95/99). HCC with better differentiation demonstrated non-rim fluorescence patterns, while cases with poor differentiation demonstrated rim patterns. CRLM uniformly demonstrated rim pattern. The width of fluorescence surrounding tumors was 0 in HCC with non-rim patterns. The minimum width of fluorescence surrounding tumors in poor differentiated HCC and CRLM were 2.4 ± 1.9 mm and 2.8 ± 2.5 mm, respectively, with no significant difference (*P* > 0.05). ICG fluorescence imaging revealed eight small lesions, which were not detected preoperatively in seven patients, of which five lesions were confirmed as malignancies by pathology.

**Conclusions:**

Resection along the ICG fluorescence edge can supply a safe surgical margin only for CRLM, but not for HCC. Otherwise, ICG fluorescence tumor imaging can preliminarily determine the pathological type of hepatic malignancies and histological differentiation of HCC and help detect small lesions that cannot be detected preoperatively.

Since the first laparoscopic hepatectomy (LH) in 1992, this technique has been increasingly used in the treatment of hepatic malignant tumors.^1^ Numerous studies have shown that LH to treat hepatic malignancies is safe and efficacious, with superior short-term and comparable long-term efficacy compared with open hepatectomy.^1,2^ Liver resection technology is constantly being improved, such as the application of intraoperative ultrasound, which is useful for detecting tumor lesions intraoperatively. However, owing to some disadvantages of laparoscopic surgery, such as the lack of palpation, it is still hard to accurately locate tumor lesions and define tumor margins.^3^ Therefore, a new imaging method is needed to help detect tumor lesions and navigate in real time to ensure safe surgical margins.

Indocyanine green (ICG) binds to proteins in the blood and emits fluorescent signals at near-infrared wavelength. ICG is taken up by hepatocytes and excreted in the bile. However, the ICG excretion process inside and around the tumor is abnormal and characterized by stagnation due to disordered excretion.^3,4^ So, in liver surgery, ICG fluorescent imaging is used in tumor detection, biliary tract contrast, and the identification of portal vein dominated areas, such as liver segmentation. Some studies have shown that ICG fluorescence imaging has advantages in intraoperative tumor visualization, but there are few reports on ICG fluorescence imaging for detecting small lesions and securing safe surgical margins.^4–6^ Recently, Tashiro et al. reported that ICG fluorescent imaging is helpful for securing a safe surgical margin in liver resection from the perspective of pathology.^7^ However, there are few reports on whether using ICG fluorescence imaging helps determining safe surgical margins in real-time navigation for laparoscopic hepatectomy.

This study retrospectively analyzed a group of patients who received ICG fluorescent laparoscopic hepatectomy to explore the value of ICG fluorescent staining of tumor in LH, especially for determining the safe surgical margin.

## Patients and Methods

### Patients

This study was approved by the institutional review board of Fujian Medical University Union Hospital and included 86 patients who underwent LH for hepatocellular carcinoma (HCC, *n* = 52), or colorectal liver metastasis (CRLM, *n* = 34) at the Department of Hepatobiliary Surgery of Fujian Medical University Union Hospital, between August 2018 and August 2021. The patient characteristics are presented in Table 1. Among the 52 cases of HCC, 50 had a single lesion and two cases had double lesions, with a total of 54 lesions. Among the 34 cases of CRLM, 26 had a single lesion, five cases had two lesions, and three cases had three lesions, with a total of 45 metastatic lesions.

**Table 1:** Patient characteristics

	HCC	CRLM
*Gender (n)*		
Male	40	22
Female	12	12
Age (mean ± SD/year)	54.7 ± 15.3	55.7 ± 9.6
*HBsAg (n)*		
Positive	49	5
Negative	3	29
*Live cirrhosis (n)*		
Positive	46	1
Negative	6	33
ICG-R15 (mean ± SD/%)	6.3 ± 3.8	3.5 ± 2 .6
*Surgical procedure (n)*		
Anatomical resection	13	0
Nonanatomical resection	39	34
*Number of lesions (n)*		
Single	50	26
Multiple	2	8
Tumor size (cm)	3.95 ± 2.94	4.30 ± 3.02
*Microvascular invasion (n)*		
Positive	12	2
Negative	40	32

*HCC* hepatocellular carcinoma, *CRLM* colorectal liver metastasis, *ICG-R15* indocyanine green clearance test

### Surgical Procedures

All patients received the assessment of liver reserve function by indocyanine green clearance test (ICG-R15) at a dose of 0.5 mg/kg peripheral intravenous (IV) ICG before surgery. Surgery was performed on day 5 after examination for patients with ICG-R15 ≤ 7%, or on day 7 after examination for patients with ICG-R15 > 7%. All patients underwent laparoscopic hepatectomy guided by ICG fluorescence fusion imaging (Stryker, MI, USA). The surgical procedures were divided into anatomic (AR) and non-anatomic (NAR) liver resection. Thirteen cases of HCC received AR including left lateral lobectomy (eight cases), left hemi-hepatectomy (three cases), right hemi-hepatectomy (one case), and right posterior lobectomy (one case). When performing the left/right hemi-hepatectomy and right posterior lobectomy, liver resection line was marked by “negative staining technique” (1.25 mg of ICG was intravenously injected after blocking the target hepatic pedicle). The other 39 cases of HCC and all the 34 cases of CRLM underwent NAR using the Pringle’s method to block the first hilar and using fluorescence detection combined with intraoperative ultrasound to detect the location and number of the lesions. Two cases (2/86, 2.33%) were transferred to laparotomy due to intraoperative bleeding.

### Measurement of Specimens

**Fig. 1:**
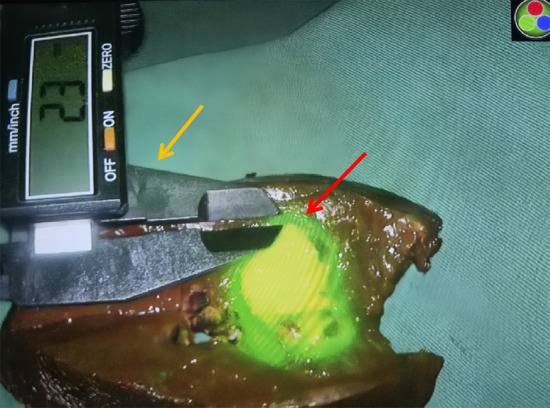
The width of the fluorescence band surrounding tumor (red arrow) measured with an electronic vernier caliper (yellow arrow)

Detection of fluorescence: the excised specimen was dissected, and the maximum diameter of the tumor was measured at the maximum section. A laparoscopic fluorescence imaging system was used to check the fluorescence staining pattern. Under the monitoring of the fluorescence imaging system, the width of the fluorescence band surrounding the tumor was measured with an electronic vernier caliper, and the minimum width was selected and recorded (Fig. 1). Pathological diagnosis: all specimens were pathologically diagnosed by two experienced pathologists. The cell type and differentiation degree of the tumor, vessel carcinoma embolus, and liver cirrhosis were also analyzed.

### Statistical Analysis

Continuous data were expressed as mean ± standard deviation (SD). Continuous data and categorical exact data were compared using the *t*-test and chi-squared test, respectively. *P*-value < 0.05 was considered statistically significant. SPSS 13.0 software was used for the statistical analysis.

## Results

### Imaging Patterns and Width of ICG Fluorescence in Different Hepatic Malignancies

**Fig. 2:**
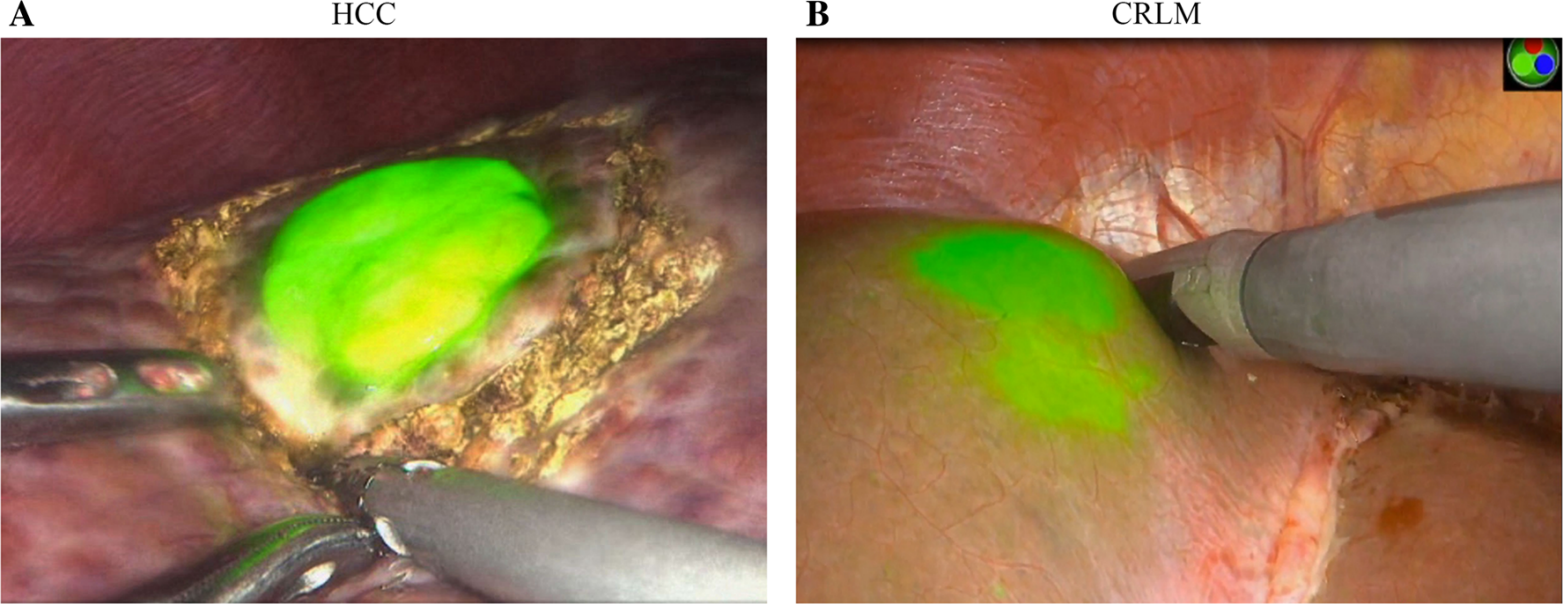
ICG fluorescence imaging of hepatic malignant lesions in HCC (**A**) and CRLM (**B**)

There were 99 malignant lesions in the 86 patients included in this study, of which 95 lesions were successfully stained by ICG fluorescence with a positive rate 96.0%. The positive staining rates of HCC and CRLM lesions were 96.3% (52/54) and 95.6% (43/45), respectively (Fig. 2).

**Fig. 3:**
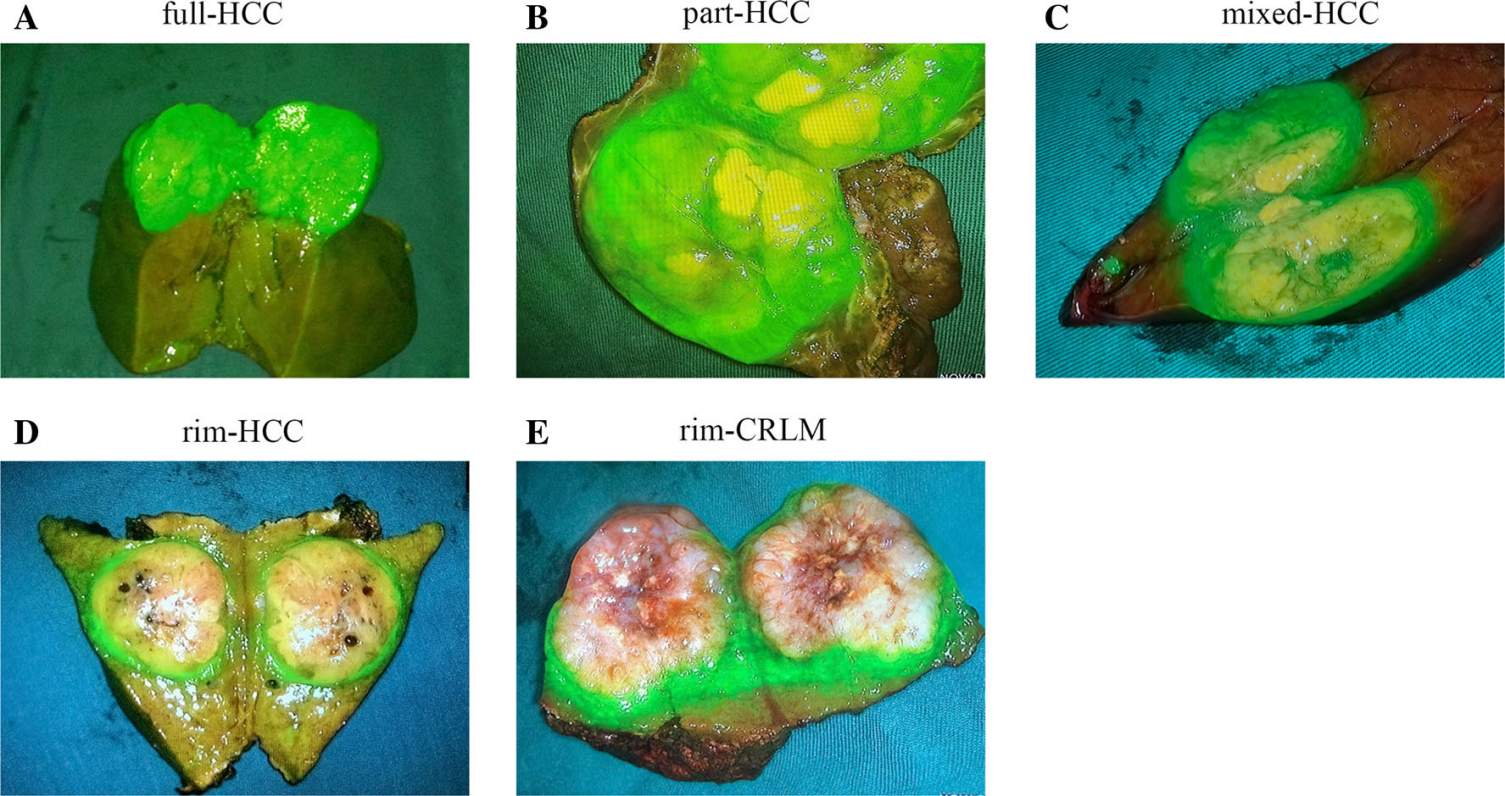
ICG fluorescence imaging patterns of hepatic malignant lesions showing the full pattern of HCC (**A**), part pattern of HCC (**B**), mixed pattern of HCC (**C**), rim pattern of HCC (**D**), and rim pattern of CRLM (**E**)

As seen from Table 2 and Fig. 3, the ICG fluorescence imaging patterns of HCC lesions can be divided into four types: total, partial, mixed, and rim patterns. The fluorescence boundary of the lesions in the non-rim (total and partial) group with HCC basically overlapped with the tumor edge, while an inhomogeneous rim fluorescence band was observed around the lesion in the rim (mixed and rim) group with HCC. All the lesions of CRLM uniformly presented rim fluorescence patterns.

**Table 2:** Imaging patterns of ICG fluorescence in different hepatic malignancies

	Non-rim patterns		Rim patterns	
	Total	Partial	Mixed	Rim
HCC^a^	12	8	9	23
I	1	0	0	0
II	11	8	0	0
III	0	0	8	19
III + IV	0	0	1	2
IV	0	0	0	2
CRLM	0	0	0	43

^a^According to the Edmondson–Steiner classification system

The average minimum width of fluorescence from the tumor edge in the non-rim group with HCC, the rim group with HCC, and the CRLM group were 0 mm, 2.4 ± 1.9 mm and 2.8 ± 2.5 mm, respectively. We further analyzed the relationship between the width of rim fluorescence surrounding tumors and pathological factors in the cases with rim fluorescence patterns. As presented in Table 3, the minimum width of rim fluorescence surrounding tumors is negatively associated with the tumor type, tumor size, liver cirrhosis, neoadjuvant chemotherapy, and *KRAS* and *BRAF* mutation (all *P* > 0.05).

**Table 3:** Relationship between the minimum width of the rim fluorescence band and pathological factors

	*n*	Minimum widths of rim fluorescence band around the tumor	*P*-value
*Tumor type*			
HCC	32	2.4 ± 1.9	0.375
CRLM	43	2.8 ± 2.5	
*Liver cirrhosis in HCC*			
Negative–mild	12	2.9 ± 2.5	0.383
Moderator–severe	20	3.5 ± 1.8	
*Tumor size of HCC*			
< 2 cm	8	2.7 ± 1.9	
2–5 cm	20	2.3 ± 2.0	0.849
> 5 cm	4	2.2 ± 1.0	
*Tumor size of CRLM*			
< 2 cm	13	2.7 ± 1.3	
2–5 cm	23	2.8 ± 3.05	0.833
> 5 cm	7	3.4 ± 0.0	
*Neoadjuvant chemotherapy for CRLM*			
Yes	37	2.8 ± 2.6	0.768
No	6	3.2 ± 2.4	
KRAS mutation in CRLM			
Positive	15	3.1 ± 1.8	0.914
Negative	19	3.0 ± 3.2	
*BRAF mutation in CRLM*			
Positive	3	2.8 ± 2.9	0.861
Negative	31	3.1 ± 2.6	

### Imaging Patterns of ICG Fluorescence in HCC

The lesions of HCC display four patterns on ICG fluorescence imaging. Therefore, we further analyzed the relationship between histological differentiation and fluorescence imaging patterns in the patients with HCC. As presented in Table 2, the better differentiated HCC (grades I and II, according to the Edmondson–Steiner classification system) showed total or partial fluorescence imaging patterns, while the poorly differentiated HCC (grades III and IV) showed mixed and rim patterns, which suggested that different fluorescence imaging patterns of HCC may be determined by the histological differentiation of cancer tissues.

Fifty-two patients with HCC were further divided into two groups: the rim (including mixed and rim fluorescence imaging patterns, *n* = 20) and non-rim group (including total and partial pattens, *n* = 32), and the relationship between the fluorescence imaging pattens and clinical factors were analyzed. The results showed that the fluorescence imaging pattern of HCC was positively correlated with the histological differentiation (*P* < 0.001), but negatively correlated with tumor size, liver cirrhosis, and hepatitis B infection (all *P* > 0.05) (Table 4).

**Table 4:** Relationship between ICG fluorescence imaging pattern and pathological factors of HCC

	Non-rim pattern	Rim pattern	*P*-value
*Histological differentiation*			
I–II^a^	20	0	< 0.001
III–IV^a^	0	32	
Tumor size (cm)	4.01 ± 2.10	3.92 ± 1.88	0.875
*Liver cirrhosis*			
Positive	18	28	0.835
Negative	2	4	
*HBsAg*			
Positive	19	30	0.274
Negative	1	2	

^a^According to the Edmondson–Steiner classification system

### The Role of ICG Fluorescence Staining in the Detection of Microscopic Lesions

ICG fluorescence staining combined with intraoperative ultrasound scan revealed three small lesions that were not detected preoperatively in three patients with HCC, and which were confirmed by pathology as hepatocellular carcinoma, nodules of cirrhosis, and bile duct adenoma, respectively. Furthermore, ICG revealed five small lesions in four patients with CRLM, of which four lesions were pathologically confirmed as metastatic carcinoma and one lesion was a fibrous nodule.

## Discussion

Indocyanine green (ICG), once bound to protein, can emit fluorescence (peaking at 840 nm) under the illumination of near-infrared light.^8^ As it can be absorbed exclusively by hepatocytes and excreted through bile without enterohepatic recirculation, ICG has gain the attention of hepatobiliary surgeons in recent years.^5^ In 2008, Aoki reported for the first time that ICG injected via the portal vein for fluorescence staining during anatomic segmental hepatectomy could clearly show the boundary plane between liver segments.^9^ In 2009, Ishizawa reported that intraoperative fluorescence imaging was useful for the determination of the tumor localization after preoperative injection of ICG via the peripheral vein.^10^ Since then, related reports have gradually increased, but the application of ICG fluorescence staining and navigation is largely unknown, and needs to be further explored.^11–13^

The ICG fluorescence imaging patterns of hepatic lesions depend on the ability of tumor cells to absorb ICG and the time difference of ICG metabolism between tumor and normal liver tissues. Therefore, the appropriate dose and preoperative administration time of ICG are crucial to ensure a good image of fluorescence staining.^14,15^ Some liver diseases such as liver cirrhosis and fatty liver, or using chemotherapy drugs weakens the metabolic function of liver, resulting in impaired metabolic capability of ICG. In these conditions, the metabolism of ICG in the body will be incomplete, which results in uneven staining of fluorescence and makes it difficult to distinguish the demarcation line between reserved and pre-cut liver. In this study, we determined the time of surgery according to the result of ICG-R15 examination (dose of ICG was 0.5 mg/kg). If the result of ICG-R15 was ≤ 7%, surgery was performed 5 days after ICG-R15 examination. If the ICG-R15 > 7%, surgery was performed 7 days after ICG-R15 examination.^16^ The positive rate of ICG fluorescence staining was 96% for all lesions, with good-quality fluorescence imaging.

In this study, we found that different types of hepatic malignancies showed different staining patterns of ICG fluorescence. The lesions of HCC with better histological differentiation showed non-rim (including total and partial) fluorescence staining patterns, while the lesions of CRLM and HCC with poor histological differentiation showed rim (including rim or mixed) patterns. It is known that cancer cells in the lesions of HCC with better histological differentiation still retain partial function of normal hepatocytes and can absorb ICG from plasma, while the dysfunction of the capillary bile duct surrounding a tumor leads to the obstruction of ICG excretion. Therefore, the lesions of well-differentiated HCC show ICG fluorescence in whole or part of the tumor. On the contrary, the cancer cells in the lesions of CRLM and HCC with poor histological differentiation have no function as hepatocytes and cannot absorb ICG, so no fluorescence can be seen in the body of these lesions. Meanwhile, the function of excreting bile of the normal tissue around the tumor is impaired due to tumor compression, resulting in ICG retention and the formation of a ring fluorescence staining pattern.^17^ In addition, due to the heterogeneity of HCC, there exists different differentiated tissues in a single lesion, resulting in the formation of a mixed fluorescence staining pattern. In general, we can preliminarily determine the tumor type and the histological differentiation of cancer tissues by the ICG fluorescence staining pattern of hepatic lesions.

The surgical margin surrounding a tumor is closely related to tumor recurrence and the patient’s survival.^18–20^ The goal of surgical treatment is to achieve a negative resection margin, but there is still no consensus on how much width of normal tissue around the tumor should be removed during hepatectomy. For CRLM, most authors believe that a resection margin of 1 mm free edge is sufficient to achieve a curative effect.^21,22^ However, the definition of surgical margin in the resection of primary liver cancers is still controversial. Due to the specific biological behaviors of HCC, such as its susceptibility to microvascular invasion, the status of the HCC resection margin is an established prognostic factor and most authors suggest a wide-margin (> 7–10 mm) hepatectomy for HCC.^23–25^ The Chinese Guidelines for the Diagnosis and Treatment of Primary Liver Cancer also suggest that the surgical margin for HCC should be greater than 10 mm away from the tumor boundary.^26^ Recent studies have shown that the use of ICG fluorescent staining of hepatic lesions may help ensure negative surgical margins.^7^ However, there have been no relevant studies on whether the criteria of surgical margin described above can be achieved or not, if there is no fluorescence exposure in the surface of the remnant liver.^27,28^

In this study, we analyzed the width of ICG fluorescence surrounding the lesions. The fluorescence imaging patterns of all lesions can be divided into two categories: non-rim fluorescence pattern (without a rim fluorescence band surrounding the lesion) and rim fluorescence pattern (with a rim fluorescence band surrounding the lesion). Non-rim fluorescence pattern appears in the lesions of better-differentiated HCC, while the rim fluorescence pattern appears in poorly differentiated HCC and CRLM, which were characterized by an uneven fluorescence band surrounding the lesion and an average minimum width of fluorescence from the tumor edge of 2.4 ± 1.9 mm and 2.8 ± 2.5 mm, respectively. Therefore, resection of the lesion along fluorescence could only provide enough and a safe surgical margin for patients with CRLM (> 1 mm), but not for those patients with HCC (especially for those cases with non-rim fluorescence imaging patterns). Therefore, we suggest combining ICG fluorescence imaging with intraoperative ultrasound to obtain a wide surgical margin in the radical resection of HCC.

Another principle for R0 resection is excision of all lesions. However, small hepatic lesions are frequently missed in diagnosis due to the absence of typical imaging manifestations, leading to failure of R0 resection and seriously affecting the prognosis. ICG fluorescence imaging has a high sensitivity for the detection of small lesions within 1 cm from the liver’s surface, which is helpful to discover small lesions that cannot be found by preoperative imaging examination. However, there still exist some shortcomings in ICG fluorescence staining for liver tumors. It was reported that the positive rate of ICG fluorescence staining for liver tumors was 43–100%.^16^ The positive rate in this study was 96.0%, indicating that some lesions of hepatic malignancies were negative for ICG staining, which would lead to a missed diagnosis. Meanwhile, hepatic benign lesions such as nodules of cirrhosis, liver cyst, and hepatic focal nodular hyperplasia can also be stained by ICG fluorescence, which results in the failure to correctly identify the nature of small lesions in the liver surface by ICG staining. In this study, the false positive rate of malignant tumor was as high as 37.5% among eight small lesions accidentally discovered by ICG staining during surgery. In addition, the penetration distance of ICG fluorescence in the liver tissue is less than 10 mm, resulting in failed detection of lesions located in the deep of liver. Therefore, we suggest that multiple imaging examination methods such as ultrasound and magnetic resonance imaging (MRI) scans should be used in combination before surgery to improve the preoperative diagnostic accuracy. Meanwhile, ultrasound scans and rapid pathological examination should be combined during surgery to remove all suspicious lesions as far as possible, to improve the R0 resection rate of liver malignancies.

## Conclusions

Our data suggest that ICG fluorescence imaging is a reliable navigation tool, which when combined with intraoperative ultrasound scanning, is helpful for tumor detection, including the identification of small lesions that cannot be detected preoperatively. Resection along the fluorescence edge can supply a safe surgical margin only for CRLM, but not for HCC. Therefore, wide margin resection should be performed as far as possible for HCC.
